# The Role of Tight Junctions in Atopic Dermatitis: A Systematic Review

**DOI:** 10.3390/jcm12041538

**Published:** 2023-02-15

**Authors:** Spyridoula Katsarou, Michael Makris, Efstratios Vakirlis, Stamatios Gregoriou

**Affiliations:** 1 1st Department of Dermatology and Venereology, Medical School, National and Kapodistrian University of Athens, Andreas Syggros Hospital, 11528 Athens, Greece; 2 2nd Department of Dermatology and Venereology, Medical School, National and Kapodistrian University of Athens, Attikon University Hospital, Allergy Unit, 12461 Athens, Greece; 3 1st Department of Dermatology and Venereology, Medical School, Aristotle University, 54124 Thessaloniki, Greece

**Keywords:** atopic dermatitis, atopic eczema, claudins, tight junctions

## Abstract

Background: Tight junctions are transmembrane proteins that regulate the permeability of water, solutes including ions, and water-soluble molecules. The objective of this systematic review is to focus on the current knowledge regarding the role of tight junctions in atopic dermatitis and the possible impact on their therapeutic potential. Methods: A literature search was performed in PubMed, Google Scholar, and Cochrane library between 2009 and 2022. After evaluation of the literature and taking into consideration their content, 55 articles were finally included. Results: TJs’ role in atopic dermatitis extends from a microscopic scale to having macroscopic effects, such as increased susceptibility to pathogens and infections and worsening of atopic dermatitis features. Impaired TJ barrier function and skin permeability in AD lesions is correlated with cldn-1 levels. Th2 inflammation inhibits the expression of cldn-1 and cldn-23. Scratching has also been reported to decrease cldn-1 expression. Dysfunctional TJs’ interaction with Langerhans cells could increase allergen penetration. Susceptibility to cutaneous infections in AD patients could also be affected by TJ cohesion. Conclusions: Dysfunction of TJs and their components, especially claudins, have a significant role in the pathogenesis and vicious circle of inflammation in AD. Discovering more basic science data regarding TJ functionality may be the key for the use of specific/targeted therapies in order to improve epidermal barrier function in AD.

## 1. Introduction

Atopic dermatitis (AD) or atopic eczema is an inflammatory pruritic dermatosis with long-standing course and relapses. Allergic rhinitis, food allergy, and asthma are Th2-driven atopic comorbidities of AD. AD affects up to 20% of children and up to 14% of adults with variations in different ethnicities and geographical regions [1]. The pathogenesis of AD is multifactorial with genetic, immunological, environmental, and microbial components. Epidermal barrier impairment has a prominent role in the pathogenesis of AD, which is attributed to filaggrin null mutations; involucrin, loricrin, and lipid (mainly ceramide) abnormalities; and interference between epidermal barrier cells and immunological pathways that entail the Th2 differentiation of lymphocytes in AD [2]. Tight junctions (TJs) are a network of transmembrane proteins, located between cells, that link to intercellular proteins. Their role is to manage the cross-talk between cells that exchange water, solutes including ions, and water-soluble molecules [3]. This review focuses on the current knowledge regarding the role of the TJs in AD, as well as possible investigational future perspectives.

## 2. Materials and Methods

**Figure 1:**
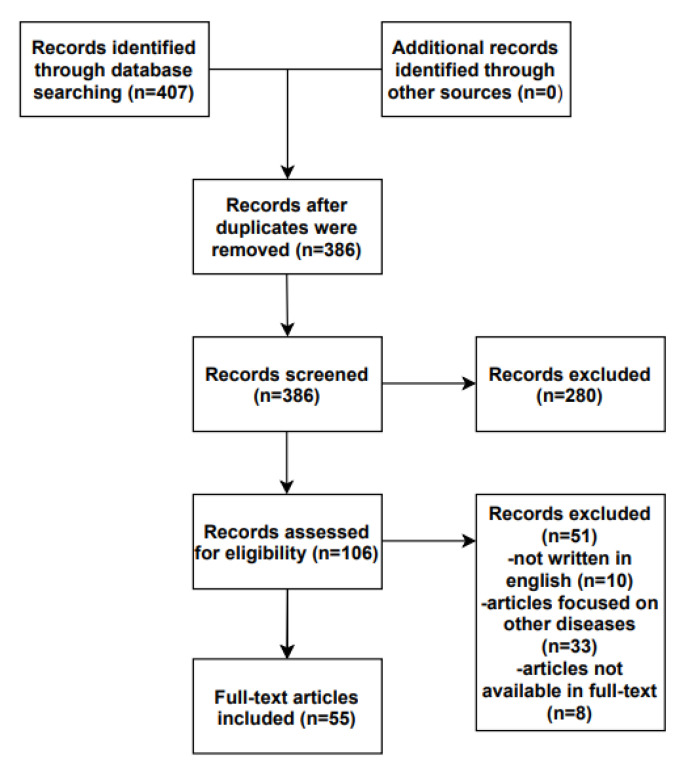
Flow diagram.

This systematic review has been performed using the PRISMA guidelines for Systematic Reviews and Meta-Analysis 2020 checklist. Literature indexed from 2009 to 2022, describing the role of tight junctions in atopic dermatitis, was searched. We identified eligible studies using the following inclusion criterion: (1) written in English. The exclusion criteria were: (1) articles focused on other diseases, such as psoriasis or ichthyosis vulgaris, in which AD was merely mentioned; (2) articles whose full-text version was not available to us; and (3) articles written in a language other than English. We performed the literature search between 2009 and 2022 in the PubMed, Cochrane Library, and Google Scholar databases, using the following terms: “atopic dermatitis” OR “atopic eczema” AND “tight junctions” OR “claudins”. Two authors individually reviewed the database search results, assessing the titles, evaluating the abstracts, and considering or not the study for full review. Any disagreements in either the title/abstract or the full manuscript review phases were resolved by consensus. All eligible studies were evaluated and included in this systematic review (Figure 1).

## 3. Results

### 3.1. The “Anatomy” of TJs

**Figure 2:**
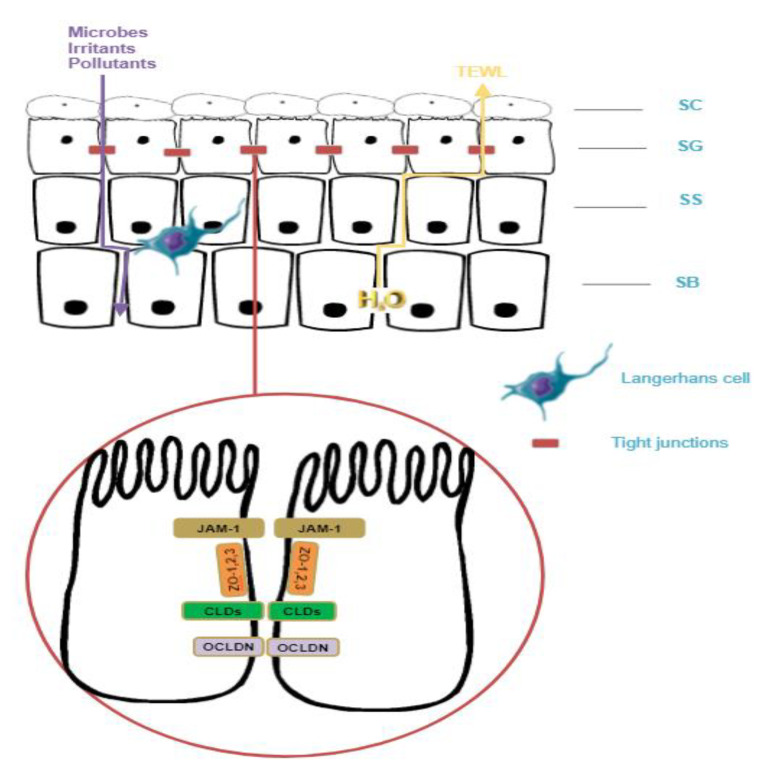
Tight junction components.(SC: stratum corneum, SG: stratum granulosum, SS: stratum spinosum, SB: stratum basale, TEWL: transepidermal water loss).

TJs are connection structures between cells that exist in simple, multi-layered epithelia and endothelia, and consist of transmembrane proteins (claudins (Cldns), junctional adhesion molecule A (JAM-A), TJ-associated marvel proteins (TAMP) (occludin (Ocln), and tricellulin) and TJ plaque proteins (e.g., the zonula occludens proteins ZO-1 and ZO-2, MUP P-1, cingulin, and symplekin) [4,5] (Figure 2).

Tjs are present in human skin, as well as in other human epithelia, such as the small intestine and blood–brain barrier. Tjs are located between endothelial cells of the cerebral microvasculature in the blood–brain barrier, controlling the homeostasis of the central nervous system. Alterations in the functionality of Tjs could lead to neurological disorders [6]. In the small intestine, Tjs form a network between the intestinal cells and impede the penetration of pathogens via the gut barrier [7].

Tight junctions in the human epidermis are composed of sets of continuous intramembranous strands, as has been revealed by freeze-fracture replica electron microscopy studies, and are found directly below the SC on opposing membranes of granular layer corneocytes [8,9]. They are primarily built by the polymerization of cldns, which are associated with the other transmembrane proteins (e.g., ocldn and tricellulin) and scaffold proteins (e.g., ZO proteins, cingulin, and membrane-associated guanylate kinase inverted proteins) [8,10].

Cldns family proteins include 27 members [11], and ocldn and ZO proteins are the principal units of TJs. The majority of them are confined to stratum granulosum (SG), although TJ proteins are also found in the stratum spinosum (SSP) (ZO-1; ZO-2; and cldns-4, -6, and -18) and stratum basale (SB). Cldn1 and cldn-12, as well as MUPP-1 and JAM-A are localized at the cell–cell borders of all living cell layers, while ocln, cingulin, and cldn-3 are limited to the SG. Cldn-7 is observed in all cell levels, with a decreased presence in SB [5,12,13,14]. Skin epithelial cells express cldn-1 and cldn-4, which mainly serve as the paracellular water barrier [11,15].

The expression of cldn-4 is high in the SG between the four levels of the epidermis, and is regulated by ΔNp63, a p53 family transcription factor, which plays a key role in epithelial differentiation. In healthy human epidermis, ΔNp63 and cldn-4 expression are inversely correlated. Distinct types of cldns and other TJ-associated molecules are expressed in all epidermis layers. Nevertheless, an effective TJ form is found only in SG, where cldn-4 is more prominently expressed [16].

#### Tight Junctions in Skin Appendages

Functional TJs are also found in hairy skin and form an effective seal between the hair and the dermis all around the keratinized club hair. TJs in human anagen hair follicles (HF) form barriers that are less dense than the scalp and cheek epidermal barriers. TJ proteins in the infundibulum and isthmus of club HF and in the outer root sheath surrounding the club hair shaft, are located in the outermost epithelial layer. Functional TJs also exist in the external layers of the outer root sheath and circle the club hair close to the keratinous rootlets. The removal of extracellular calcium leads to TJ opening and to disruption of the HF barrier. Cldn-1 is more abundant than other TJ proteins in anagen and club HF, and its expression in HFs is crucial for the HF barrier functionality and for hair growth [17]. A TJ structure is also found in the paracellular space of luminal cells of the sweat gland apparatus, forming a barrier to prevent body fluid leakage [18]. Luminal cells in the sweat gland apparatus express Cldn-3, which in turn prevents the drainage of sweat in murine sweat gland apparatus. A reduction in cldn-3 levels can lead to increased sweat drainage in a dose-dependent manner. The stinging sensation while sweating in AD could be attributed to sweat drainage. Cldn’s specific roles have been described in the human sweat glands, such as the role of cldn-10 and cldn-15 in sodium absorption regulation [19].

TJ-regulated permeability also affects the selective diffusion of molecules from deeper layers to the surface of the barrier. Injecting a 557 Da molecular weight tracer subcutaneously in a rat model of normal and cldn-1-deficient mice revealed that the tracer could only pass through TJs to the skin surface in the cldn-1-deficient mice. This supports the hypothesis that Tjs control the paracellular movement of multiple molecules and in multiple directions within the barrier [20].

### 3.2. The Role in Healthy Skin

TJs contribute to many cellular functions, such as differentiation, polarity, proliferation and signaling cascade processes [4]. Their role in homeostasis includes the formation of junctions between cells in order to connect them, controlling the exchange of molecules and separating the different parts of the cell membranes [21]. They recruit cytoskeletal and signaling molecules and regulate epidermal permeability [8]. They form a barrier against the diffusion of solutes through the intercellular space and their defects lead to the penetration of irritants, microbial products, toxins, and allergens. This was initially reported in mice lacking cldn-1 that presented high trans-epidermal water loss and significant loss of function of the skin barrier [20]. They are independently regulated in each organ, as cldns expressed in the skin (cldn-1, cldn-4, cldn-12, cldn-25) are different from those expressed in other organs such as the lung, where cldn18 and JAM-C are uniquely found [22]. TJ function is also affected by Toll-like receptor (TLR) signaling. TJs respond to internal signals that arise from epithelial tissues and to external signals such as bacterial-derived products and allergens, in a very dynamic way, by loosening and tightening. Staphylococcus aureus has also been proposed to affect Tj characteristics through TLR signaling [23].

### 3.3. Tight Junctions in Atopic Dermatitis

Dysfunctional TJs contribute to irregular function of the skin barrier in AD. Disrupted TJs appear to impede SC formation through an increase in the SC potential of hydrogen (pH), failure to mature lamellar structures, and degradation of keratohyalin granules. Damaged TJ barriers can affect the processing of polar lipids and profillagrin by disturbing the pH condition of SC [24]. The TJ barrier of the skin is not directly affected by filaggrin deficiency. The literature suggests that filaggrin knockout mice present no alterations in TJ morphology, TJ expression, or TJ barrier function. The available data suggest that cutaneous inflammation is the main driver for epidermal TJ barrier dysfunction. Subsequently, TJ impairment results in SC damage and in increased permeability of exogenous bacterial and allergen molecules, leading to a vicious circle of barrier dysfunction and skin inflammation [25].

#### 3.3.1. Claudins Regulation in Atopic Dermatitis

Studies show that hereditary or acquired cldn-1 defects can be seen in AD subjects [26]. A study in two North American populations showed that in the African/American population, there was a strong association between an intronic single nucleotide polymorphism (SNP) with a reduced risk of AD (rs17501010) and a neighboring SNP with an increased risk of AD (rs9290927). SNP-rs17501010 also appeared to be associated with the early onset of AD (<5 years of age) in African/Americans. Two additional SNPs (rs893051 in intron 1 and rs9290929 in the promoter region) were associated with greater disease severity in the same population. On the other hand, in the European/American population a promoter SNP (rs16865373) seemed to be related with a lower risk for AD and a lower risk of early onset AD (<5 years of age). Intriguingly, in a Northern European population, the same cldn-1 SNPs (rs893051, rs9290927, rs9290929, and rs17501010) were related to contact allergens hypersensitivity [12]. Another study found that rs893051 in a native Ethiopian population was associated with early onset AD [27]. It has also been reported that patients with the rs9290929 polymorphism, located in the cldn-1 promoter, who were exposed to mold during the first year of life, had an independent risk factor for lifetime AD symptoms when compared with children with the same polymorphism but no mold exposure [28].

Cldn-1 has been suggested to control AD features time- and dose-dependently [15]. A reduction in cldn-1 expression levels is a critical risk factor for human AD. In the elderly, reduced levels of cldn-1 expression cause hyperkeratosis and acanthosis. Exponential correlations between the expression level of Cldn1 and its epithelial barrier function and with the phenotype have been reported in animal experimental models. More severe phenotypes have been observed in Cldn1 mutant mice with an extremely low expression of Cldn1, near the threshold for lethality. In addition, age-dependent changes in their skin appearance have been observed, including wrinkled skin at 1 week, abnormal dry hair at 2 weeks, and nearly normal skin at 8 weeks [15]. In Cldn-1-deficient mice that died 24 h after birth due to dehydration, the epidermal barrier was damaged, although keratinocyte organization appeared to be normal. The immunofluorescence staining of SG showed that cldn-1 and cldn-4 were continuously concentrated in the cell–cell boundaries. This led to the conclusion that continuous cldn-based TJs occur in the epidermis and are crucial in epidermal barriers [20]. Another study with cldn-1-deficient mice showed that they exhibited abnormally wrinkled and rough corneocytes, a tracer easily penetrated the SC, and water evaporation was significantly high. In addition, SC lipid ceramide composition and fillagrin processing were reported to be abnormal [20,29,30].

In AD, cldn-1 is downregulated in both lesional and non-lesional skin. However, cldn-1 downregulation is not observed in healthy skin. Downregulation of cldn-1 has been reported to be caused by inflammation and the degree of downregulation is analogous to the density of the inflammatory infiltrate in the lower and upper levels of the epidermis [31]. De Benedetto et al. identified a reduced expression of cldn-1, 4, 23, and 25 in clinically unaffected AD skin of a Northern American cohort, which could contribute to the increased accessibility of this skin to allergens and flares of atopic dermatitis lesions [12,32,33]. Other studies have reported a decrease in cldn-1 expression in non-lesional and lesional skin and an up-regulation in cldn-4 and ocldn in non-lesional skin, and downregulation (cldn-4 in SG) and upregulation (Cldn-4 in uSSP and mid SSP (mSSP), ocln in all layers) in lesional skin has also been reported [34].

Impaired TJ barrier function and skin permeability in AD lesions is correlated with cldn-1 levels [34]. Cldn-1 shows a dose-dependent upregulation of IL-1β, with the subsequent infiltration of inflammatory cells [31,32,34]. However, another study reported a significant down-regulation of cldn-1 in different epidermal layers of lesional AD skin, and no down-regulation of cldn-1 or up-regulation of cldn-4 in non-lesional skin of AD patients [35]. Finally, other researchers observed a downregulation in cldns 1, 5, 11, and 23, as well as a significant downregulation in cldns 4 and 8 in lesional compared with non-lesional and normal epidermis [36]. These results may indicate that different populations or subset populations may have genetic differences concerning cldn-1. An exonic mutation in cldn-1 can cause a human syndrome, called neonatal ichthyosis-sclerosing cholangitis. These patients have features similar to AD (erythema, dry flaky skin, and patchy alopecia) and severe liver and gallbladder abnormalities that are likely due to the importance of cldn-1 in the integrity of the bile duct barrier [32].

Regarding ZO-1, reduced synthesis is found in non-lesional and lesional AD skin [37]. Cldn-1 has also been observed to be remarkably down-regulated in the HFs of lesional but not non-lesional skin in AD patients compared with in healthy participants [17]. As HFs act as the gate of entrance for drugs and chemicals, TJs might be the primary fencing in skin appendages without SG. Consequently, the absence of TJs in AD enables the entrance through HFs of antigens and viruses such as herpes simplex or molluscum contagiosum. The permeability of the epithelium due to TJ dysfunction in HFs and non-follicular skin, which is induced by the Th2 immune response, leads to a vicious cycle of antigen-driven activation of the innate and adaptive immune system [22].

TJ damage is significant in the vicious cycle of itching and scratching. Scratching has also been reported to decrease the cldn-1 expression without affecting ZO-1 or ocldn expression, and to increase Akt phosphorylation in AD [38]. The Erk and Akt signaling pathways are essential for the formation and preservation of TJs. An agonist of Akt phosphorylation also reduces cldn-1 expression, so the inhibition of Akt phosphorylation can rescue cldn-1 expression and reduce scratching [38].

#### 3.3.2. The Role of P63

P63 is a regulator with multiple roles in cutaneous development and differentiation, including regulation of the junction complexes between cells within the epidermis. In AD, p63 is overexpressed and this is related to pruritus, dry skin, and high IgE. P63 can directly lead to the activation of IL-31 and IL-33, which are the main components of Th2 inflammation [39]. The ΔNp63-deficient keratinocytes of AD skin highly express cldn-4. P63 is a negative regulator of cldn-4 expression in primary keratinocytes. Several components can regulate Cldn-4 in normal epithelial cells and diseases. In human keratinocytes and nasal epithelial cells, p63 (TAp63 and ΔNp63) can directly regulate Cldn-4. Downregulation of ΔNp63 through treatment with short interfering RNA-p63 induces the expression of cldn-4 [40]. Consequently, p63 may act as a target for therapeutic approaches, as its role in AD seems to be of great importance.

#### 3.3.3. Host Defense Peptides and Tjs

Host defense peptides, or antimicrobial peptides, are involved in various biological procedures, such as chemotaxis promotion, the production of cytokines and chemokines, dendritic cell and macrophage differentiation, neutrophil and epithelial cell apoptosis, pro-inflammatory response suppression, angiogenesis, and wound healing induction. Cathelicidin LL-37, human β-defensins, and S100 protein psoriasin (S100A7) participate in skin defense. In skin, keratinocytes and neutrophils transport LL-37 when they infiltrate infected or wounded skin. Normally, the expression of LL-37 is scarcely detectable in keratinocytes. On the other hand, during infection or injury, its production is strongly increased. Human cathelicidin LL-37 is overexpressed in lesional psoriasis, but decreased in lesional AD. It has been found that LL-37 selectively increases the expression and membrane distribution of TJ proteins and is involved in enhancing TJ barrier function through the activation of aPKC, Rac1, GSK-3, and PI3K signaling pathways. Thus, in addition to its antimicrobial and immunomodulatory activities, LL-37 contributes to skin immunity through regulation of the skin barrier function [21].

#### 3.3.4. Increased Susceptibility to Infections

AD patients are more vulnerable to cutaneous infections than non-AD patients. Widespread HSV-1 infections may be associated with a reduced cldn-1 expression and a subsequently destroyed TJ function [41]. Viruses use the cell–cell proteins in order to infect. In particular, wild-type HSV-1 can destroy human keratinocytes through a reaction that involves the viral envelope glycoprotein gD with either nectin-1 (cell surface molecule that belongs to the immunoglobulins class) or the HSV entry mediator. Nectin-1, which is Ca-independent, collocated with E-cadherin and β-catenin, creates the adherens junction (an intercellular junctional complex). The human keratinocytes are vulnerable to HSV-1 infection in a manner negatively associated with the extent of intercellular proximity [9].

AD skin is significantly more colonized by *S. aureus* than normal skin. This colonization leads to reduced functionality of coagulase-negative staphylococci, which are part of the normal skin microbiota spectrum and exert a protective effect against pathogens. *S. aureus* expresses proteins that contribute to epidermal barrier dysfunction and superantigens that promote pro-inflammatory cytokine production [42]. However, investigational data show a short-term promoting effect on the TJ barrier initially followed by subsequent impairment through dysfunction of the Toll-like receptor 2, which is essential for TJ cohesion in the case of pathogen attack. Dysfunction of TJ cohesion in AD subjects has been associated with increased vulnerability to bacteria and skin infections [23]. Normally, when human epidermal keratinocytes have contact with *S. aureus*, the TJ barrier function increases. Later, however, the TJ barrier function decreases. This biphasic effect causes TJ protein relocation but not quotative TJ protein level alterations [43].

#### 3.3.5. Langerhans Cells and TJs

Dendritic cells (DCs) are white blood cells that mediate antigen presentation and protection against microbes. Langerhans cells (LCs), which are a DCs’ subclass, function in the human epidermis beneath the TJ barrier, establishing a complex system. Activated LCs have dendrites that invade into the TJs of the healthy human epidermis [3]. During SC disruption, LCs dendrites penetrate TJs in order to attach to the antigens and protect against antigens that tend to break through the skin barrier. This function of TJs is regulated by toll-like receptors.

TJ activity changes depending on the external environment, showing a dynamic management in order to protect against the invasion of external microorganisms [37]. Although both LCs and inflammatory dendritic epidermal cells (IDECs) express FcεRI below the TJs, IDECs do not extend their dendrites through the TJ [3]. In AD, the number of LCs that penetrate TJs is increased, and this probably leads to an increased allergen uptake [31]. The number of DCs in the epidermis of patients with AD is analogous to the allergen burden and severity of AD phenotype and inversely proportional with clinical improvement [3]. DCs express CD206 and higher levels of FcεRI than LCs [3]. DCs produce proinflammatory cytokines that induce a TH1 response and lead to the chronic phase of AD [3]. LCs produce smaller amounts of proinflammatory cytokines and induce a TH2 T-cell response [3].

Langerin is a type of pattern recognition receptor and more specifically a C-type lectin receptor that is expressed only in LCs and takes part in host defense against many viruses, fungi, and bacteria. Dendrites of activated LCs present Langerin in AD skin, while DCs do not [3]. Langerin interaction with *S. aureus* is strongly associated with skin inflammation in AD [44].

#### 3.3.6. Inflammatory Cytokines and TJs

Inflammation, especially Th2 inflammation, inhibits the expression of important cldns (cldn-1 and cldn-23). A negative correlation exists between epidermal cldn-1 expression and markers of Th2 polarity [12]. Th17 cells infiltrate AD skin lesions, especially during the acute phase of dermatitis. Th17 cells produce IL-17, which decreases TJ function through the inhibition of ZO-1, cldn-1, and cldn-4 protein synthesis in AD. This has been reported to adjust epithelial cells in the local “environment” and trigger keratinocytes to produce chemokines and cytokines [37]. After skin is exposed to IL-17, the ocldn expression is altered from a continuous to an intermittent way in SG [37]. Furthermore, IL-17 downregulates the expression of the filaggrin gene; however, profilaggrin synthesis has been reported to be decreased by the Th2 cytokines IL-4 and IL-13. IL-17 promotes increased thickness of the cutaneous horny layer and abnormalities in filaggrin degradation and TJ function [37]. IFN-γ, a Th1 cytokine highly expressed in the lesional skin of chronic AD, decreases the expression of the cldn-1 protein in a dose- and time-dependent manner. This suggests that the downregulation of the cldn-1 expression causes IFN-γ-mediated disruption of TJ function [45]. Cldn-4 expression has been reported to increase by IL-17A, while cldn-1 has not. This increase was found to be prevented by co-treatment with IL-4 [46]. Finally, IL-33 down-regulates the expression of cldn-1 in human keratinocytes [47]. Th2 cytokines also down-regulate the expression of cldn-1 and, by extension, TJs, as well keratins, filaggrin, and desmosomal cadherins. This leads to the disruption of keratinocyte differentiation, barrier disruption, and the promotion of type 2 mediators such as thymic stromal lymphopoietin (TSLP), IL-25, and IL-33. These cytokines trigger basophils, innate lymphoid cells type 2 (ILCs), and DCs, and are involved in type 2 inflammation. ILC2s can directly destroy TJs [22]. In addition, it has been suggested that type 2 inflammation might have a synergistic effect with fillagrin deficiency [48]. TJs have also been reported to dysfunction in psoriasis. ZO-1 and occluding are found in a more expanded zone from the granular layer to the middle spinous cell layers in psoriatic plaques, but distribution returns to normal in ameliorated psoriatic lesions [49]. An abnormal occluding expression has been observed after both IL-17 and TNF-alpha exposure [50,51].

#### 3.3.7. TJs and Potential Therapeutic Interventions

The literature suggests that several agents could exert pharmacological modification of TJs. Spirodela polyrhiza extract (SPE) and OLE (Olea europaea leaf extract) have positive effects on AD symptoms. Treatment with both OLE and SPE as a combined mixture has been reported to improve pruritus and epidermal hyperplasia, stabilize the immune response (reduction of the inflammatory and allergenic cell infiltration and cytokine levels), reduce the levels of IgE and histamine, and restore skin barrier function (up-regulation of expression of skin barrier proteins) [52]. Aquaphilus dolomiae extract-G1 has been reported to modulate the inflammatory response (stimulates IL-8 and reduces CCL20 expression), augment the activities of antimicrobial peptides (stimulates DEFB4 and A100A7) and enhance barrier function through the restoration of filaggrin expression [53]. Topical application of a high concentration of glucose has been suggested to reduce skin inflammation and induce cldn-1 and filaggrin expression in inflamed skin [54]. Cimifugin remarkably diminishes allergic inflammation by a reduction of TSLP and IL-33 production by TJ regulation. Thus, it might ameliorate the junctions’ deficiency between epithelial cells, rehabilitate the expression of epithelial TJs, and inhibit the epithelial derived initiative key factor [55]. PAG (DL-Propargylglycine) inhibits keratinocyte proliferation, restores TJ expression, and also suppresses the production of pro-inflammatory cytokines in AD skin lesions [56]. The JAK kinase inhibitor, delgocitinib, improves TJ dysfunction as a result of IFN-γ [45]. Topical application of 0.5% ointment, once daily, does not induce skin atrophy or decreased immunohistochemical staining of claudins. This is in opposition with the commonly used topical corticosteroids, which decrease cldn-1 and -4 expression, modify epidermal TJ components, and cause damage to the skin barrier function [57]. Proteasome inhibitors, especially bortezomib, improve AD symptoms in mice. The degradation of cldn-1 is made through the ubiquitin–proteasome pathway, so these inhibitors tend to increase the expression of cldn-1 in human keratinocytes and skin [58]. Topical corticosteroids decrease the expression of genes and proteins of TJ components, such as Cldn-1 and -4 and Ocln, and consequently modify TJ structures. Topical calcineurin inhibitors, on the other hand, do not modify the gene and protein expression of Cldn-1 and -4, but decrease the expression of ocln. Ocln does not seem to be crucial for barrier function, in contrast with cldn-1 and -4, which are very essential. Consequently, as topical corticosteroids downregulate the expression of Cldn-1 and -4, they are able to affect the permeability of the TJ barrier in a negative manner. Conversely, topical tacrolimus application may affect the function of the TJ barrier in a positive way, it preserves Cldn-1 and -4 [59]. Systemic treatment also has a theurapeutic effect on the TJ component. Increased immunochemistry lesional skin staining of Cldns has been reported after nbUVB, cyclosporine, and dupilumab treatment [60,61].

### 3.4. Future Perspectives

As more data on TJs role in AD become available, unanswered questions to be further investigated emerge. The effect of filaggrin expression downregulation on TJs functionality is an area that needs further investigation. Exploring the crosstalk between TJs and the immune system is also on the forefront of current research. Developing topical agents that improve TJ functionality, among other action modes, is an emerging therapeutic field. Pivotal clinical trials now include genomics data, which might help to illuminate basic unanswered science questions on AD pathogenesis, including the potential of TJs.

### 3.5. Limitations

TJs in AD is an emerging subject in the literature. This means that the publication of new data could possibly change our current knowledge as presented in this review in the near future. In addition, only literature written in English was considered. As a result, literature written in another language may have been skipped.

## Data Availability

Data sharing not applicable.

## Abbreviations

AD	atopic dermatitis
TJs	tight junctions
Cldn	claudin
JAM-A	junctional adhesion molecule A
TAMP	TJ-associated marvel proteins
Ocln	occluding
ZO	zonula occludens
SC	stratum corneum
SB	stratum basale
SG	stratum granulosum
SSP	stratum spinosum
HF	hair follicles
SNP	single nucleotide polymorphism
DC	Dendritic cell
LC	Langerhans cell
